# Genetic Diversity and Interpopulation Variability of the Hong Kong Newt (*Paramesotriton hongkongensis*) in an Urbanized and Deforested Landscape

**DOI:** 10.1002/ece3.70849

**Published:** 2025-04-07

**Authors:** Anthony Lau, Shu‐Ping Tseng, Nancy E. Karraker, David Dudgeon

**Affiliations:** ^1^ Science Unit, School of Interdisciplinary Studies Lingnan University Hong Kong SAR China; ^2^ Department of Entomology National Taiwan University Taipei City Taiwan, ROC; ^3^ Center for Applied Fire and Ecosystem Science New Mexico Consortium Los Alamos New Mexico USA; ^4^ School of Biological Sciences The University of Hong Kong Hong Kong SAR China

**Keywords:** gene flow, habitat fragmentation, population genetics, reforestation, Salamandridae, urbanization

## Abstract

Population genetics is a valuable tool for conservationists to quantify population‐level genetic variation and identify priority conservation units. The Hong Kong newt ( *Paramesotriton hongkongensis* ) is a tropical salamander restricted to streams and forests in southern China, facing significant challenges from range‐wide deforestation since the 1600s, and recent rapid urban development. Using species‐specific microsatellite markers, we found surprisingly high genetic diversity within and among *P. hongkongensis* populations, despite long‐term habitat disturbance and fragmentation. Only 2 out of 10 sites exhibited evidence of recent population bottlenecks. Bayesian clustering revealed four well‐supported genetic clusters within the newt's Hong Kong range, suggesting that these should be managed as separate conservation units. Our findings highlight the resilience of this species to historical and contemporary disturbances and emphasize the importance of considering genetic data in conservation planning for amphibians in human‐modified landscapes.

## Graphical abstract

In this paper, we investigated the genetic diversity and population genetic structure of an endangered species of salamander using species‐specific microsatellite markers. Our results indicated that the remaining populations are still genetically diverse and protected areas in place are effective in maintaining both genetic diversity and structure. However, management authorities should consider the genetic origins of animals in any future translocation/relocation efforts.

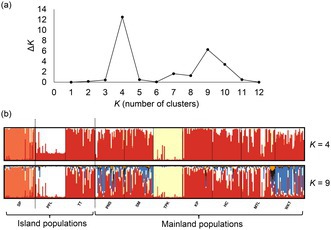

## 1 Introduction

Genetic diversity, defined as a measure that quantifies the genetic variability within a population, is a fundamental source of biodiversity. Preserving natural genetic diversity, which represents adaptive potential of a population, is important in the context of rapid and sometimes irreversible environmental change, including destruction of habitat, invasive species, disease outbreaks, and climate change (Crozier 1997; Hoban et al. 2013; DeWoody et al. 2021). In addition to loss of fitness in inbred populations (i.e., inbreeding depression) (Lacy 1993; Caro and Laurenson 1994; Keller and Waller 2002), attrition of genetic diversity also theoretically limits a population's ability to withstand and adapt to changing environmental conditions as the global climate changes (Gienapp et al. 2008; Merilä and Hendry 2014). Environmental change may especially impact animals with limited dispersal capabilities, such as amphibians, which are experiencing steep global population declines (Luedtke et al. 2023). Unfortunately, little is known about the genetic variation within and among populations of many amphibians, or the ecological and evolutionary patterns influencing such variation, hindering efforts to conserve them.

In addition to natural landscape features such as mountains and rivers which shape population structure over geologic time scales (Lougheed et al. 1999), contemporary (past 200 years) anthropogenic disturbances may also affect the genetic structure of amphibian populations, particularly in highly modified landscapes where remnant patches of forest still harbor isolated populations of amphibians (Zellmer and Knowles 2009; Noël and Lapointe 2010; Munshi‐South, Zak, and Pehek 2013). Such effects of contemporary fragmentation on population differentiation may even override those of historic fragmentation (Zellmer and Knowles 2009). In Europe, loss of genetic diversity and reduced fitness have been reported in fragmented populations of several widespread anuran species (Rowe and Beebee 2003; Johansson, Primmer, and Merilä 2007; Luquet et al. 2011). In contrast, two species of narrowly distributed frogs in China still maintained a relatively high level of genetic diversity, despite their threatened status (Zhang et al. 2015; Pan et al. 2019). In the face of ongoing global amphibian declines (Blaustein, Wake, and Sousa 1994; Stuart et al. 2004), understanding intrapopulation differentiation is needed to conserve vulnerable amphibian species in highly fragmented habitats where natural dispersal and genetic exchange are disrupted by urban development (Noël and Lapointe 2010; Munshi‐South, Zak, and Pehek 2013). Nevertheless, a recent analysis of the effects of urbanization on the genetic diversity of 19 amphibian species in North America failed to find any generalizable positive or negative effects between genetic metrics and human disturbance (Schmidt and Garroway 2021).

**FIGURE 1:**
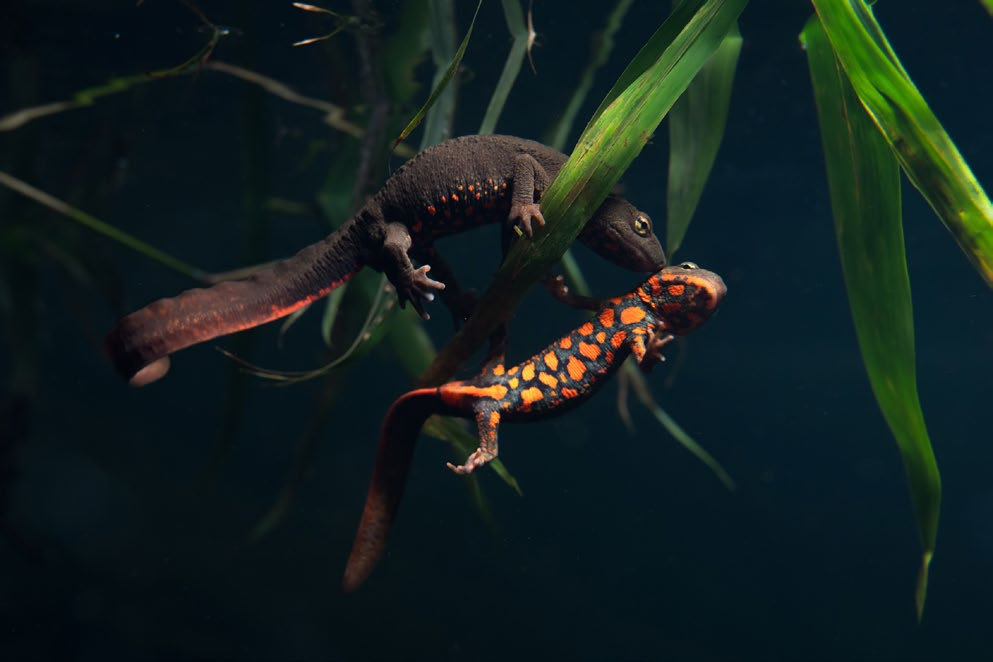
Adult Hong Kong newts (*Paramesostriton hongkongensis*) in situ with sweet flag ( *Acorus gramineus* ), which they use as oviposition sites. Photo by Hon Shing Fung.

**FIGURE 2:**
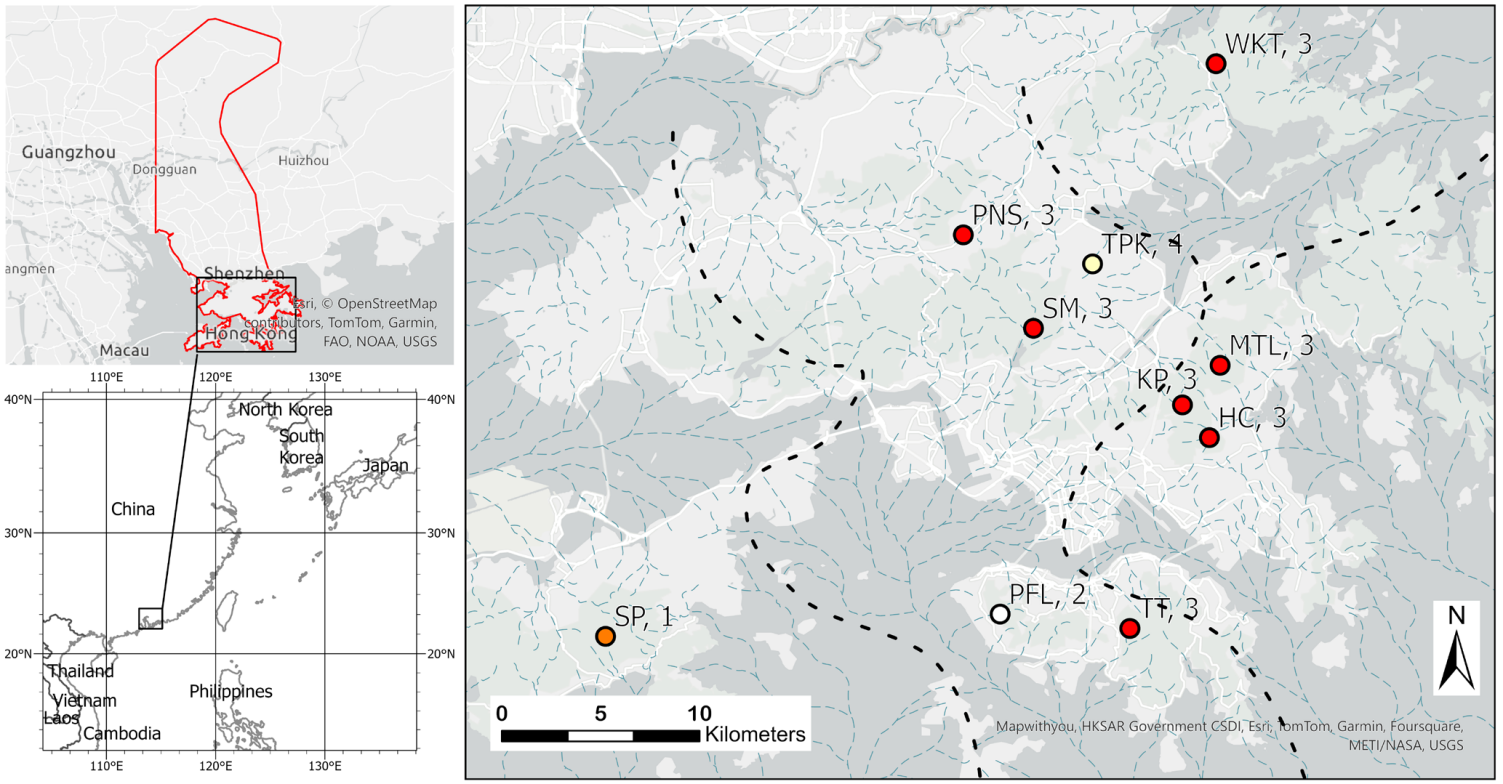
Geographic distribution (left panel, in red) and sample collection sites (right panel) of *Paramesotriton hongkongensis* in HKSAR, 2013–2014. Site codes as in Table 1, and color and number of site symbols represent cluster memberships from STRUCTURE. Light blue dashed lines represent late Pleistocene paleodrainage pattern and black dashed lines represent paleodrainage basins adopted from Wong et al. (2017). HI, Hong Kong Island; LI, Lantau Island; NT, Mainland New Territories.

The Hong Kong newt ( *Paramesotriton hongkongensis* ) is a tropical salamander that inhabits small rocky hill streams and riparian forests in coastal Guangdong Province, southern China (Figure 1). In the Hong Kong Special Administrative Region (HKSAR), populations of *P. hongkongensis* occur on the mainland New Territories and on two outlying islands (Lau and Chan 2004; Figure 2). Outside of Hong Kong, it is found in at least three mountain ranges in southern China, which are up to 30 km away from the Hong Kong‐China border (IUCN 2020; Figure 2). The species is classified as “Near Threatened” on the IUCN Red List due to its small extent of occurrence (EOO, < 20,000 km^2^), habitat degradation throughout its range, and vulnerability to overexploitation for the pet trade (Lau and Chan 2004; IUCN 2020). Notably, from 2005 to 2010, over 200,000 *P. hongkongensis* , presumably adults from the wild, were exported from Hong Kong to the United States (Kolby et al. 2014). Locally, it is one of only three amphibian species listed under the Wild Animals Protection Ordinance (Cap. 170), which prohibits hunting, disturbance, and possession of this animal without a special permit issued by the Agriculture, Fisheries and Conservation Department of Hong Kong.

Populations of *P. hongkongensis* in HKSAR tend to be isolated from each other as they occur on both the mainland New Territories and adjacent major islands in a topographically complex, highly fragmented landscape where there are many potential barriers to dispersal (e.g., roads, urban development, and sea) (Zhang et al. 2011). In addition to contemporary, range‐wide habitat fragmentation caused by urban development, the habitat of *P. hongkongensis* also experienced large‐scale deforestation starting as early as the 1600s when most forested habitats were repeatedly logged (Dudgeon and Corlett 2011). As forest cover has been found to be positively related to gene flow for several salamander species (Emaresi et al. 2011; Richardson 2012; Emel and Storfer 2015), reduction in forest cover from logging and subsequent fragmentation of remnant habitats could lead to reduced gene flow and increase the probability of genetic drift in populations of *P. hongkongensis* in HKSAR.

The goals of our study were to (1) quantify population genetic diversity and (2) characterize the population genetic structure of *P. hongkongensis* using species‐specific microsatellite markers. Since populations of *P. hongkongensis* have experienced repeated and ongoing habitat disturbance in the form of deforestation and urbanization throughout the species' range, we expect genetic diversity to be low due to historic population declines. We also expect population structure to largely be shaped by geographic features such as river systems and dispersal barriers such as the sea.

## 2 Materials and Methods

### 2.1 Field and Molecular Methods

We collected samples from 10 second‐ or third‐order streams (classification following Strahler 1957) that contained breeding pools of *P. hongkongensis* in HKSAR from August 2013 to January 2014 (Figure 2). Of these 10 streams, seven streams are located on the mainland New Territories and three streams (PFL, TT, and SP) are located on islands. We considered each of our streams as individual populations because these streams are in different drainage basins and the minimum distance between sites (~2 km) far exceeded the maximum reported terrestrial displacement of *P. hongkongensis* (~0.4 km, Fu, Dudgeon, and Karraker 2013; Lau et al. 2017). Because of the concerns over illegal collection for the wildlife trade, precise locations are not provided but are available upon request.

Tail clips (~2 mm) were taken from 310 *P. hongkongensis* , preserved in 95% ethanol, and stored at −20°C before DNA extraction. Total genomic DNA was extracted using FavorPrep Tissue DNA Extraction Mini Kit (Favorgen Biotech Corp., Ping‐Tung, Taiwan) following the manufacturer's protocol. Nine polymorphic microsatellite loci developed for *P. hongkongensis* (*Phon07*, *Phon16*, *Phon18*, *Phon23*, *Phon24*, *Phon36*, *Phon48*, *Phon60*, and *Phon65*; Lau, Karraker, and Dudgeon 2016) were amplified by polymerase chain reaction (PCR) using fluorescently labeled (6FAM, NED, PET, or VIC) forward primers and conditions described in Lau, Karraker, and Dudgeon (2016). These primers were developed using a liver tissue sample collected from an individual from the KP population, which is in the central portion of the species range within HKSAR. While the markers were developed from a single population, they were selected based on successful cross‐population amplification rather than polymorphism levels in the source population. The similar levels of allelic richness observed across populations (see Section 3) suggest minimal impact of any potential ascertainment bias from the marker development process.

PCRs were performed in a 10 μL reaction volume containing 5 μL 2× GoTaq colorless master mix (Promega Corp., Madison, Wisconsin, USA), 0.2 μM labeled forward primer, 0.2 μM reverse primer, 3 μL ddH_2_0, and 1 μL genomic DNA (~50 ng/μL). Reaction conditions were: 5 min at 95°C, followed by 30–35 cycles of 95°C for 30 s, 48°C–62°C for 30 s, and 72°C for 30 s, with a final extension at 72°C for 10 min. Resulting PCR products were electrophoresed on a 3130xl Genetic Analyzer (Applied Biosystems) with GeneScan LIZ 500 (Life Technologies) as an internal size standard, and manually scored and aligned using *GeneMapper* v4.0 (Applied Biosystems). We used *MicroChecker* v2.2.3 (van Oosterhout et al. 2004) to check for scoring errors, allelic dropouts, and null alleles.

### 2.2 Population Genetic Analyses

We used *Genepop* (v4.3; Rousset 2008) to calculate number of alleles (Na), calculate expected and observed heterozygosity (H_E_ and H_O_, respectively), and test for linkage disequilibrium (LD) and deviation from the Hardy–Weinberg equilibrium (HWE). We used *FSTAT* (v2.9.3.1; Goudet 2001) to calculate allelic richness. We compared measures of genetic diversity among sites using the non‐parametric Kruskal–Wallis *H* test implemented in R (ver 3.1.1).

*BOTTLENECK* v1.2.02 (Piry, Luikart, and Cornuet 1999) was used to test for the presence of recent population bottlenecks within each site (Luikart and Cornuet 1998). We performed a Wilcoxon signed‐rank test for heterozygosity excess in each site using the stepwise mutation model (SMM), which allows for variation in percentage of multi‐step mutation and is the suggested model for testing recent bottlenecks using microsatellite data (Luikart and Cornuet 1998). Following Peery et al. (2012), we set the mean size of multi‐step mutations to 3.1 and proportions of multi‐step mutations to 0.12, 0.22, and 0.32, and ran each test with 10,000 permutations.

The Bayesian clustering program *STRUCTURE* (ver2.3.4; Pritchard, Stephens, and Donnelly 2000) was used to infer population structure of *P. hongkongensis* . *STRUCTURE* infers population structure by assigning individuals to different genetic clusters, and the number of clusters (*K*) reflects the level of population subdivision (Pritchard, Stephens, and Donnelly 2000). We ran STRUCTURE from *K* = 1 to *K* = 12 (total number of sampling sites +2) with five independent runs for each *K*, using the admixture model with uncorrelated allele frequencies. For each run, we ran 1 million Markov Chain Monte Carlo repetitions with the initial 100,000 generations discarded as burn‐in, following Gilbert et al. (2012). We determined the best K using the delta K method (Evanno, Regnaut, and Goudet 2005) implemented in *STRUCTURE Harvester* (Earl 2009). We used *CLUMPP* (ver1.1.2; Jakobsson and Rosenberg 2007) to convert output from *STRUCTURE* and *CLUMPAK* to visualize the output (Kopelman et al. 2015).

*Arlequin* (v3.5; Excoffier and Lischer 2010) was used to calculate and test the significance of pairwise genetic distance (F_ST_) between sampling sites. We also performed an analysis of molecular variance (AMOVA) using *Arlequin* to partition total genetic variance into the within populations, among populations within the main genetic clusters as determined by *STRUCTURE*, and among clusters components. The number of permutations for F_ST_ analysis and AMOVA were 10,000 and 1000, respectively.

Lastly, to examine the effects of geographic distance on genetic distance between sites (i.e., isolation‐by‐distance), we conducted a Mantel test (IBDWS v3.23; Jensen, Bohonak, and Kelley 2005), which tested for significant correlations between pairwise genetic distances (*F*_ST_) and geographic distances. We used Euclidean distances between sites measured in *ArcMAP* (ver10.2; ESRI, Redlands, CA, USA) and *F*_ST_ values calculated from *Arlequin*. We used 10,000 permutations to assess significance.

## 3 Results

### 3.1 Genetic Diversity

A total of 310 individuals from 10 sites across HKSAR (mean *N* = 31, SD = 1.563, range: 30–35) were genotyped. No evidence of allelic dropout and scoring error was found. No loci deviated significantly from HWE following sequential Bonferroni correction (Rice 1989) or showed evidence of LD. Null alleles were detected in *Phon24* and *Phon48* and, thus, these loci were excluded from further analysis. The remaining seven microsatellites revealed high genetic diversity per population (Table 1), with an allelic richness of each locus ranging from 8.5 to 14.1 (mean = 13.0, SD = 2.1), observed heterozygosity ranging from 0.631 to 0.814 (mean = 0.748, SD = 0.088), and expected heterozygosity ranging from 0.706 to 0.890 to (mean = 0.839, SD = 0.030). Average values for genetic diversity measures were similar across sites, and there were no statistically significant differences in Na, A_R_, or H_O_, although values from PFL, SP, and TPK were slightly lower. H_E_ values were also similar among study sites, with significantly lower values at TPK only (Table 1). Two (PNS and SM) out of ten sites showed evidence of population bottlenecks (Table 2).

**TABLE 1:** Measures of genetic diversity among seven microsatellite loci from 310 *Paramesotriton hongkongensis* collected from 10 sites across HKSAR, 2013–2014.

Site	Sample size	Number of alleles (Na)	Allelic richness (A_R_)	Observed heterozygosity (H_O_)	Expected heterozygosity (H_E_)
HC	30	13.9	13.4 ± 4.5	0.788 ± 0.125	0.863 ± 0.060
KP	31	14.4	13.7 ± 3.6	0.805 ± 0.178	0.860 ± 0.060
MTL	35	15.0	13.8 ± 3.2	0.704 ± 0.103	0.851 ± 0.071
PFL	31	10.7	10.2 ± 4.8	0.788 ± 0.125	0.863 ± 0.060
PNS	30	14.6	14.1 ± 4.1	0.814 ± 0.097	0.890 ± 0.034
SM	30	14.7	14.1 ± 4.7	0.810 ± 0.096	0.885 ± 0.036
SP	32	10.9	10.4 ± 1.9	0.763 ± 0.108	0.816 ± 0.063
TPK	30	8.9	8.5 ± 2.1	0.631 ± 0.143	0.706 ± 0.124
TT	31	12.7	12.0 ± 3.5	0.783 ± 0.108	0.852 ± 0.039
WKT	30	13.9	13.3 ± 4.1	0.752 ± 0.186	0.873 ± 0.028
Kruskal–Wallis		*χ* ^2^ = 14.08	*χ* ^2^ = 14.76	*χ* ^2^ = 13.10	*χ* ^2^ = 23.98
test statistics		*df* = 8 *p* = 0.08	*df* = 8 *p* = 0.06	*df* = 8 *p* = 0.11	*df* = 8 *p* = 0.002

*Note:* Average values (± SD) across loci for each site are reported.

**TABLE 2:** Results from Wilcoxon sign rank test for heterozygote excess performed in *BOTTLENECK* ver1.2.02.

Site	Bottleneck *p* value		
	% SMM = 0.12	% SMM = 0.22	% SMM = 0.32
HC	0.29	0.66	0.47
KP	0.66	0.98	0.97
MTL	0.77	0.98	0.98
PFL	0.66	0.97	0.96
PNS	0.01*	0.04*	0.04*
SM	0.01*	0.04*	0.02*
SP	0.47	0.85	0.77
TPK	0.97	0.99	0.99
TT	0.59	0.81	0.77
WKT	0.41	0.71	0.71

Abbreviation: SMM, stepwise mutation model.

* Significant *p* value at *α* = 5. Site codes as in Table 1.

Pairwise *F*_ST_ values ranged from 0.003 (PNS and SM) to 0.172 (SP and TPK) (mean = 0.067, SD = 0.038). All but one (i.e., PNS and SM) were significantly larger than 0 following Bonferroni corrections (Table 3), indicating a moderate level of genetic differentiation among populations. TPK exhibited high genetic differentiation relative to other populations, as demonstrated by *F*_ST_ values ranging from 0.107 to 0.172. Isolation‐by‐distance among sites was not significant (*R* ^2^ = 0.08, *p* = 0.16).

**TABLE 3:** Pairwise genetic (*F*_ST_; below diagonal) and geographic (in km; above diagonal) distances among *Paramesotriton hongkongensis* collected from 10 sites across HKSAR, 2013–2014.

Site	HC	KP	MTL	PFL	PNS	SM	SP	TPK	TT	WKT
HC		2.07	3.67	13.21	15.37	9.88	29.96	10.30	10.29	17.64
KP	**0.019**		2.66	29.96	13.35	7.95	29.39	8.24	11.51	16.13
MTL	**0.038**	**0.024**		16.20	13.68	8.92	31.81	7.84	13.89	13.99
PFL	**0.050**	**0.079**	**0.066**		19.15	14.46	18.50	18.13	6.11	28.61
PNS	**0.033**	**0.030**	**0.047**	**0.063**		5.74	26.22	6.22	21.27	14.54
SM	**0.035**	**0.024**	**0.038**	**0.076**	0.003		25.31	4.25	15.74	15.23
SP	**0.085**	**0.082**	**0.092**	**0.132**	**0.062**	**0.060**		29.48	24.53	40.21
TPK	**0.127**	**0.107**	**0.112**	**0.143**	**0.105**	**0.104**	**0.172**		18.41	10.99
TT	**0.050**	**0.037**	**0.046**	**0.071**	**0.033**	**0.032**	**0.099**	**0.107**		27.62
WKT	**0.045**	**0.053**	**0.056**	**0.075**	**0.021**	**0.026**	**0.070**	**0.127**	**0.057**	

*Note: F*_ST_ values in bold were significant following sequential Bonferroni correction. Geographic distances are Euclidian distances. Site codes as in Table 1.

### 3.2 Population Structure and Gene Flow

**FIGURE 3:**
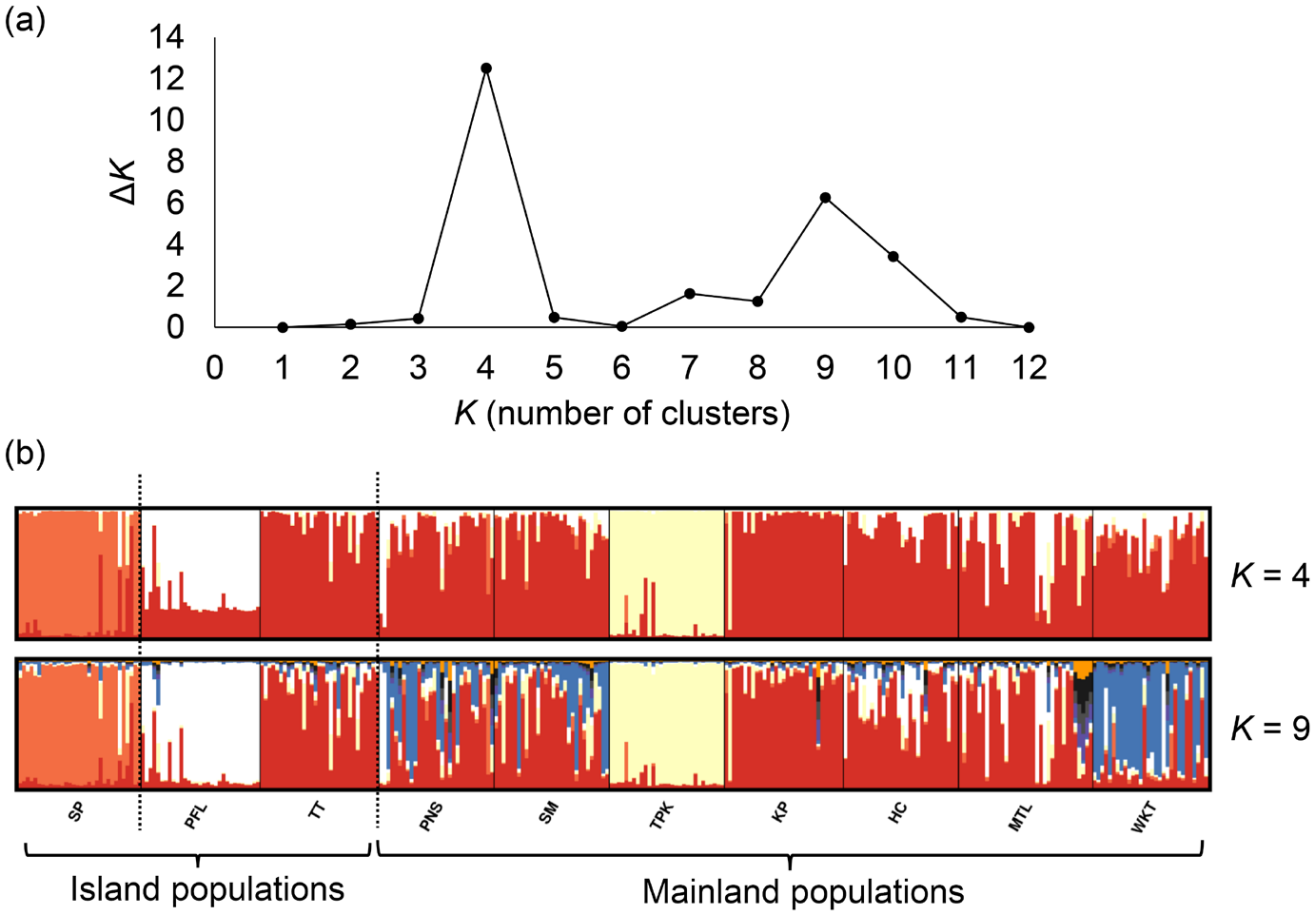
Population genetic structure of *Paramesotriton hongkongensis* as determined by Bayesian clustering. (a) Δ*K* as a function of number of clusters (*K*). (b) Bar plots for *K* = 4 and *K* = 9 showing probability of cluster assignment of each individual, arranged by site. Black dashed lines indicate sea barriers between sites. Site codes as in Table 1.

Our Bayesian clustering analysis conducted through STRUCTURE revealed that the optimal partitioning of clusters was *K* = 4 (Figure 3a). Cluster 1 comprised only the SP population, located on one of the highest peaks on Lantau Island. Cluster 2 contained only the PFL population, on the western side of Hong Kong Island. Cluster 3 included six populations sampled from the mainland New Territories and one from Hong Kong Island, whereas Cluster 4 comprised the TPK population only (Figures 2 and 3b).

Most populations showed some evidence of admixture, suggesting gene flow and shared ancestry among these clusters (Figure 3b). There was also some support for *K* = 9, which largely resembles *K* = 4, except that the northeastern most population (WKT) formed its own cluster (Figure 3b). AMOVA results showed that most genetic variation was observed within sites (92.3%) rather than among clusters (2.0%) or within clusters (5.7%) (Table 4).

**TABLE 4:** Results from analysis of molecular variance (AMOVA) conducted in *Arlequin* for seven microsatellite loci among 10 populations and four genetic clusters of *Paramesotriton hongkongensis* .

Source of variation	*df*	Sum of squares	Variance component	Percentage of variation
Among clusters	3	79.65	0.0818	1.99
Within clusters	6	111.53	0.2361	5.74
Within sites	610	2313.71	3.7930	92.3
Total	619	2503.89	4.1109	
*F*_ST_ = 0.0773***				
*F*_SC_ = 0.0586***				
*F*_CT_ = 0.0220				

*Note:* Statistical significance (*p*): ***p < 0.001.

## 4 Discussion

### 4.1 Genetic Diversity

Contrary to our prediction, *P. hongkongensis* populations exhibited high genetic diversity despite long‐term habitat disturbance and fragmentation. This finding contributes to the growing evidence that the effects of urbanization and human disturbance on genetic diversity in amphibians are not easily generalizable and should be evaluated on a case‐by‐case basis (Schmidt and Garroway 2021).

In contrast, anthropogenic disturbance and fragmentation have been associated with lower levels of genetic variability and signatures of population bottlenecks in a few species of plethodontid salamanders (Noël and Lapointe 2010; Apodaca, Rissler, and Godwin 2012; Munshi‐South, Zak, and Pehek 2013). The most likely explanation for this discrepancy is that declines in *P. hongkongensis* populations may not have been severe enough to lead to high levels of inbreeding and genetic drift. Although little is known about the historical abundance of *P. hongkongensis* , recent work on its demography shows that three out of four populations monitored from 2007 to 2014 were stable or growing (MTL, KP, and PNS; Lau, Karraker, and Dudgeon 2017). Other possible explanations for the lack of negative effects of disturbance on genetic diversity include historical levels of genetic diversity, long generation times, random mating, and lack of recent significant bottlenecks (Allentoft and O'Brien 2010).

### 4.2 Comparison With Previous Study

Our study, using microsatellite markers, revealed some contrasting results to the previous work by Zhang et al. (2011), which used amplified fragment length polymorphism (AFLP) markers. In that study, two mainland New Territories populations (PNS and TSS; the latter is referred to as SM in this study) separated by a mountain ridge were genetically more distinct from each other than either was from an island population (TTT, referred to as TT in this study) separated by a sea barrier of no more than 2 km that has existed for about 6000 years, which suggests the mountain ridge barrier had a stronger isolating effect than the sea barrier between mainland New Territories and the islands (Zhang et al. 2011). The authors also speculated a negative correlation between genetic diversity and elevation since the lower elevation site they sampled had the highest genetic diversity and vice versa. However, the small number of sites sampled (three) limits the inferences that can be drawn. In addition, the appropriateness of using AFLP markers for population differentiation has been questioned (Campbell, Duchesne, and Bernatchez 2003; Schlötterer 2004).

We included all three populations sampled by Zhang et al. (2011) in our study, which allowed us to reveal interesting parallels and contrasts with their work. In our study, the genetic distance between the two mainland New Territories sites sampled by Zhang et al. (2011) (i.e., PNS and TSS/SM) was the lowest observed (Table 3), starkly contrasting what they had found. Also, there was no positive or negative correlation between site elevation and measures of genetic diversity. Our use of microsatellite markers versus Zhang et al.'s AFLP markers might capture different aspects of genetic variation, leading to divergent results. This emphasizes the need for careful consideration of genetic markers in population studies. Microsatellite markers (as used here) represent a better alternative for investigating intrapopulation genetic structure because they are highly diverse, co‐dominant, and the results are highly repeatable when compared with AFLP markers (Jehle and Arntzen 2002; Balloux and Lugon‐Moulin 2002; Schlötterer 2004; Camacho‐Sanchez et al. 2020). However, we acknowledge that a limitation of our study was the number of markers used, which was slightly lower than similar studies of other amphibians (e.g., 10 loci in *Rhyacotriton variegatus* ; Emel and Storfer 2015).

Compared with hynobiid and plethodontid salamanders which exhibit limited movement capabilities and dependence on specific microhabitats (e.g., decaying logs and burrows), *P. hongkongensis* is capable of long‐distance dispersal nearly 0.4 km from the nearest stream and can persist in habitats with little to no canopy cover (Lau et al. 2017). In this study, a substantial portion of genetic diversity was observed within populations, which is expected for polymorphic microsatellites. Another factor that may contribute to high within‐population diversity could be the high breeding site fidelity of *P. hongkongensis* : adults migrate seasonally between the land and stream pools for breeding, with known individuals returning to the same breeding pool year after year (Fu, Dudgeon, and Karraker 2013; Lau et al. 2017). However, while most individuals recaptured during a long‐term population study were found in the same breeding pools, a small proportion could have migrated to other breeding pools during some breeding seasons (Lau, Karraker, and Dudgeon 2017). Inter‐pool and inter‐stream movements are likely to be important factors in maintaining genetic diversity in *P. hongkongensis* populations, but it is not known what factors dictate whether an individual would revisit the same breeding pool in consecutive breeding seasons, nor how such behavior contributes to the maintenance of genetic diversity of this newt.

### 4.3 Population Structure and Gene Flow

Our results indicate a complex population structure with evidence of both genetic differentiation and gene flow among populations. Bayesian clustering identified four well‐supported genetic clusters, which partially conform to major topographic features and sea barriers to dispersal across HKSAR. This genetic structuring suggests that despite the species' limited distribution and close association with streams, distinct genetic units may have evolved in response to local environmental conditions or historical isolation.

The population genetic structure of *P. hongkongensis* is considerably less complex than that observed in three other sympatric but fully‐aquatic freshwater species in Hong Kong, an atyid shrimp (*Caridina cantonensis*; Yam and Dudgeon 2005; Ma et al. 2021), a goby ( *Rhinogobius duospilus* ; Tsang et al. 2016; Wu et al. 2016), and a loach ( *Oreonectes platycephalus* ; Wang, Reid, et al. 2024). Wong et al. (2017) studied two stream‐dwelling loaches, *Schistura fasciolata* and *Pseudogastromyzon myersi* , in Hong Kong and demonstrated that their population differentiation is mainly driven by late Pleistocene paleodrainage pattern. They identified six distinct lineages in *S. fasciolata* and three in *P. myersi* , which generally corresponded to the paleodrainage basins. These lineages included Pok Fu Lam (western Hong Kong Island), Central (central mainland), East (eastern mainland and Hong Kong Island), North (northeastern mainland), Lantau, and Southwest (mainland) for *S. fasciolata* . While the number of clusters we identified in *P. hongkongensis* was the same as the number of major paleodrainage basins, the spatial arrangement of these clusters did not match the arrangement of the paleodrainage basins as seen in the loaches. In *P. hongkongensis* , the two island clusters (Clusters 1 and 2) matched with the Pok Fu Lam and Lantau lineages, while the two mainland clusters (Clusters 3 and 4) would fall under either the Central or East lineages. These discrepancies are probably due to the capacity for movement among palaeodrainages by these animals (Bohonak 1999). *P. hongkongensis* is capable of overland dispersal nearly 0.4 km (Lau et al. 2017), whereas movement by freshwater shrimp and fishes must take place within the stream or river network, thus they are more isolated within paleodrainages (Ma et al. 2021; Wang, Zeng, et al. 2024, Wang, Reid, et al. 2024). A study of two sympatric stream‐breeding salamanders in North America demonstrated that variation in life‐history traits and consequential differences in overland dispersal strongly influenced population genetic structure. The species with greater overland dispersal showed no population‐level structure across the study area while the other showed high levels of genetic structure (Steele, Baumsteiger, and Storfer 2009). In our study, half of the population structure identified (i.e., two out of four clusters) can be attributed to isolation by a sea barrier to dispersal.

The assignment of the TT and TPK populations is particularly intriguing, as neither clustered with the other populations located on the same island or landmass. TT was assigned to the cluster that contained most of the populations on the mainland New Territories, and TPK was assigned to its own cluster. There is a possibility that these populations originated from results of translocated individuals from other clusters, including those outside of our sampling locations in HKSAR (e.g., neighboring Shenzhen in southern China). In the past, the government has released captive‐bred freshwater fishes (Wong et al. 2017) and turtles confiscated from poachers (Y.H. Sung, pers. comm.) into the country parks as a conservation measure. Translocation of *P. hongkongensis* has also taken place as part of the mitigation for construction work on streams. These types of translocations are poorly documented and there is local guidelines on the translocation process. Moreover, the public frequently releases unwanted pets or practices Buddhist mercy releases, which has resulted in the establishment of feral populations of several introduced species.

While distinct clusters were identified, we also observed evidence of admixture between these clusters. This admixture suggests ongoing or recent gene flow between populations, indicating that the clusters are not completely isolated from each other. Gene flow (global *F*_ST_ = 0.067) of *P. hongkongensis* populations examined here did not appear to be highly restricted despite apparent isolation and dispersal barriers between some of the clusters and sites. Interestingly, we did not find a significant correlation between genetic distance and geographic distance among populations. The absence of a clear isolation‐by‐distance pattern suggests that factors other than simple geographic separation may be influencing the genetic structure of *P. hongkongensis* populations. Future work on *P. hongkongensis* should explore the role of landscape features, such as landcover type and slope, and physiological constraints that are known to influence gene flow among salamander populations (Wang, Savage, and Shaffer 2009; Dudaniec et al. 2012; Peterman et al. 2014; González‐Fernández et al. 2019). An alternative explanation for the lack of correlation between genetic and geographic distances could be the low number of sampled sites (only 10). If possible, future work should also focus on incorporating more populations into the analysis.

Compared to their European and North American counterparts, Asian salamanders are poorly understood but are at risk from overexploitation and environmental change or degradation. For example, more than 65% of salamanders in East and Southeast Asia are threatened (AmphibiaWeb 2024), with one species ( *Hypselotriton wolterstorffi* ) considered to be extinct (IUCN 2020). Given that many of these species have recently been described (e.g., *Hypselotriton oolong*; Wang, Zeng, et al. 2024) and are often confined to a small distribution or a single locality, there is an urgent need to assess the genetic diversity of their populations and inform conservation planning. The microsatellite markers employed here and the additional 17 recently described can be used to accomplish that, as they can be cross‐amplified in at least some East Asian species in the genus *Paramesotriton*, *Pachytriton*, and *Cynops* (Lau, Karraker, and Dudgeon 2016; Mao et al. 2024).

### 4.4 Conservation Implications

An understanding of the population genetic structure of an endangered species is essential to prioritize where to spend limited conservation funds and may also aid in the design and regulation of protected areas. Based on the results presented here, *P. hongkongensis* populations are not constrained by low genetic diversity. However, any future relocation, translocation, or reintroduction efforts should consider the genetic makeup of the individuals that will be translocated or reintroduced. Relevant agencies should consider using the genetic clusters identified here to delimit conservation management units (Dolgener et al. 2012; Sarasola‐Puente et al. 2012). For conservation interventions such as managed translocation or introductions, the four clusters identified should be regarded as distinct management units, although connectivity between the two mainland clusters should not be restricted by future land developments or other human‐associated disturbance. A particular issue not addressed in the present study is the role of roads as barriers to dispersal and gene flow. Each year, thousands of *P. hongkongensis* are killed by vehicles on roads that traverse their terrestrial non‐breeding habitat. This likely limits gene flow among populations, although the precise impacts have yet to be evaluated.

## 5 Conclusion

Our findings reveal population structure and gene flow in a tropical stream‐breeding salamander. The species exhibits distinct genetic clustering yet also shows evidence of admixture and high within‐population diversity. This pattern suggests a history of population connectivity that may have been affected by more recent fragmentation events. These results underscore the importance of developing conservation strategies that maintain existing connections between populations and preserve each cluster's unique genetic composition. By balancing these objectives, we can better ensure the long‐term viability and adaptive potential of *P. hongkongensis* populations in the face of ongoing environmental changes and habitat fragmentation.

## Author Contributions

**Anthony Lau:** conceptualization (equal), data curation (equal), investigation (equal), methodology (equal), writing – original draft (lead), writing – review and editing (lead). **Shu‐Ping Tseng:** data curation (equal), formal analysis (equal), investigation (equal), methodology (equal), software (equal), writing – review and editing (equal). **Nancy E. Karraker:** conceptualization (equal), funding acquisition (equal), project administration (equal), resources (equal), supervision (equal), writing – review and editing (equal). **David Dudgeon:** conceptualization (equal), funding acquisition (equal), project administration (equal), resources (equal), supervision (equal), writing – review and editing (equal).

## Conflicts of Interest

The authors declare no conflicts of interest.

## Supporting information


Data S1.

## Data Availability

The data that supports the findings of this study are available in the Supporting Information of this article. However, we opted not to disclose the exact locations of our study sites due to concerns over illegal collection. Please contact the corresponding author directly as needed.
